# Protective effect of influenza vaccination on cardiovascular diseases: a systematic review and meta-analysis

**DOI:** 10.1038/s41598-020-77679-7

**Published:** 2020-11-26

**Authors:** Moein Zangiabadian, Seyed Aria Nejadghaderi, Mehdi Mirsaeidi, Bahareh Hajikhani, Mehdi Goudarzi, Hossein Goudarzi, Masoud Mardani, Mohammad Javad Nasiri

**Affiliations:** 1 School of Medicine, Shahid Beheshti University of Medical Sciences, Tehran, Iran; 2 Department of Medicine, Division of Pulmonary, Critical Care, Sleep and Allergy, University of Miami, Coral Gables, Florida, USA; 3 Department of Microbiology, School of Medicine, Shahid Beheshti University of Medical Sciences, Tehran, Iran; 4 Infectious Diseases and Tropical Medicine Research Center, Shahid Beheshti University of Medical Sciences, Tehran, Iran

**Keywords:** Diseases, Health care, Medical research, Pathogenesis, Risk factors, Signs and symptoms

## Abstract

Cardiovascular diseases (CVDs) are among the leading causes of mortality and morbidity worldwide. There are many contrasting ideas on the effectiveness of influenza vaccination on CVDs. This study aimed to investigate the association between influenza vaccination and the risk of CVDs. We systematically searched all PubMed/Medline, EMBASE, and the Cochrane library entries up to November 2019 for studies of influenza vs. the CVDs outcomes. We conducted a random-effects meta‐analysis using the inverse variance method for pooled risk ratios (RR) or odds ratios (OR) and evaluated statistical heterogeneity using the I^2^ statistic. We identified 17 studies (6 randomized controlled trial [RCT], 5 cohorts, and 6 case–control) with a total of 180,043 cases and 276,898 control participants. The pooled RR of developing CVDs after influenza vaccination in RCT studies was 0.55 (95% CI 0.41–0.73), which was significant (P-value = 0.00). The pooled OR of decreasing CVDs after influenza vaccination in cohort studies was 0.89 (95% CI 0.77–1.04). The pooled OR of developing CVDs after influenza vaccination by pooling case–control studies was 0.70 (95% CI 0.57–0.86, (P-value = 0.00). All of these studies suggest decreased risks of CVDs with influenza vaccination. The current study does support the protective role of influenza vaccination on CVDs events. Health authorities may develop evidence-based preventive strategies to offer influenza vaccination in patients with CVDs.

## Introduction

Influenza vaccination is one of the most effective preventive strategies against influenza infection^1^. There are two common types of vaccines, including inactivated influenza vaccines (IIV) and live attenuated influenza vaccines (LAIV). The IIVs include two groups, trivalent (IIV3) and quadrivalent (IIV4), depending on the number of strains they contain. The IIV3 contains an H1N1 virus, an H3N2 virus, and a B virus. The LAIV, like IIV4, is a quadrivalent vaccine that contains A and B viruses^2^. There are strong recommendations for influenza vaccination in European and North American countries, where six months and older should be vaccinated annually^3^.

Cardiovascular diseases (CVDs) defined as ischemic heart disease, cerebrovascular ischemic disease (the most common type of stroke), heart failure, arrhythmia, heart valve problems, and peripheral vascular disease are a leading cause of mortality and morbidity in the world^4^.

There are many contrasting ideas on the effectiveness of influenza vaccination on CVDs events. On the one hand, some observational studies demonstrate a positive relationship between influenza vaccination and a reduced incidence of cardiovascular events such as acute myocardial infarction (AMI)^5,6^. On the other hand, some epidemiological studies indicate that influenza vaccines do not have much effect^7^.

A comprehensive study has not been conducted in recent years, so we conducted a systematic review and meta-analysis in patients with cardiovascular events such as AMI, atrial fibrillation, and stroke. The review compared outcomes in patients who had been vaccinated against influenza versus those who had not been vaccinated against influenza to investigate the risk of cardiovascular events. Thus. The current study aimed to investigate the association between influenza vaccination and the risk of CVDs.

## Methods

This review conforms to the “Preferred Reporting Items for Systematic Reviews and Meta-Analyses” (PRISMA) statement^8^. The protocol was registered in the PROSPERO database (pending registration ID: 214862).

### Search strategy and study selection

A search of the English medical literature was conducted using Medline (via PubMed), EMBASE, and the Cochrane Controlled Register of Trials (CENTRAL) from January 1, 2000, to November 23, 2019. Clinical studies investigating the relationship between influenza vaccination and subsequent risk for the development of CVDs in patients aged 18 years old or older were selected. We included randomized controlled trials (RCT), cohort studies, and case–control studies that were written in English. We used the following MeSH terms: “‘influenza vaccines’ and ‘myocardial infarction’” (Table S1). Keyword searches were done with combinations of the terms “influenza”, “flu”, “respiratory infection”, “myocardial infarction”, “cardiovascular”, “atherosclerosis”, “atrial fibrillation”, “stroke” and “coronary”. Lists of references of selected articles and relevant review articles were hand-searched to identify further studies. Title and abstracts of the articles identified by the initial search were independently evaluated by two authors and any disagreements were resolved by the lead investigator. Then, all potentially relevant articles were obtained and evaluated in detail. Articles were assessed independently by 2 investigators and any disagreements between them were resolved by discussion. The primary outcome assessed was the occurrence of CVDs in patients receiving vaccination in comparison to patients without any vaccination. The exclusion criteria were: conference abstract, case report, studies comparing high and low doses of influenza vaccination, and studies investigating predictors of influenza vaccination uptake among adults with a history of a heart attack.

### Data extraction

All data were extracted by the lead investigator to a Microsoft Excel spreadsheet (XP professional edition; Microsoft Corp, Redmond, WA). The following data were extracted: first author, country of origin, type of study, inclusion period, the definition of case and control, disease type, and the total number of controls and cases.

### Quality assessment

The checklists provided by the Joanna Briggs Institute (JBI) for cohort studies^9^, case–control studies^10^, and RCTs^11^ were used to perform the quality assessment.

### Statistical analysis

Pooled results were expressed as the risk ratios (RR) or odds ratios (OR) of patients with vaccination compared with no vaccination, with 95% confidence intervals (CIs). Each meta-analysis was performed separately for randomized controlled trials, cohort studies, and case–control studies. For each meta-analysis, the method of Der Simonian and Laird was used^12^. According to this method, studies were considered a random sample from a population of studies. Statistical heterogeneity was tested for each analysis by the I^2^ statistic. Due to the heterogeneity among studies, a random-effect model was used to analyze data. Publication bias was assessed statistically using Begg’s test (p < 0.05 was considered indicative of statistically significant publication bias). All analyses were performed using Comprehensive Meta-Analysis software, Version 2.0 (Biostat, Englewood, NJ).

## Results

**Figure 1:**
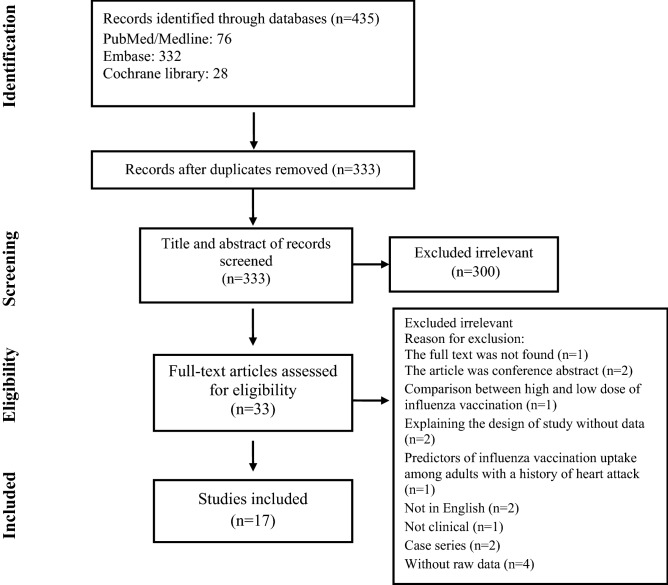
Flow chart of study selection for inclusion in the systematic review and meta-analysis.

Of the 435 articles identified, 17 studies met the inclusion criteria (Fig. 1). The characteristics of the included articles are summarized in Table 1. Among included studies: 6 articles had RCT, 5 articles had a cohort, and 6 articles had case–control designs. Based on JBI, all included studies had a low risk of bias. In the RCT articles, there were 3677 cases and 3681 controls among the whole population. There were 78,522 cases and 127,833 controls in the cohort studies and 97,844 cases and 145,384 controls in the case–control studies.

**Table 1:** Characteristics of included studies.

Study design	First author	Published year	Country	Age range	Matching	Definition of case (number of participants)	Definition of control	Outcomes
RCT	Dokainish^32^	2019	Multicenter	≥ 18	1:1	Vaccinated patients with heart failure and NYHA functional class II, III, and IV (n = 2500)	Unvaccinated patients with heart failure and NYHA functional class II, III, and IV (n = 2500)	Cardiovascular death, non-fatal myocardial infarction, non-fatal stroke, and hospitalizations for heart failure
	Kuanprasert^5^	2011	Thailand	≥ 65	1:1, sex, age	Vaccinated patients with the acute coronary syndrome (n = 221)	Unvaccinated patients with the acute coronary syndrome (n = 218)	Coronary ischemic events, stroke, heart failure, and vascular death
	Bilińska1^33^	2010	Poland	51–69	1:1, sex, age	Vaccinated patients with stable angina (n = 242)	Unvaccinated patients with stable angina (n = 259)	Coronary ischemic events
	Bilińska2^33^	2010	Poland	51–67	1:1, sex, age	Vaccinated patients with acute coronary syndrome (n = 83)	Unvaccinated patients with acute coronary syndrome (n = 74)	Coronary ischemic events
	Bilinska3^34^	2008	Poland	32–80	1:1, sex, age	Vaccinated patients with coronary artery disease (n = 335)	Unvaccinated patients with coronary artery disease (n = 333)	Coronary ischemic events
	de la Fuente1^23^	2004	Argentina	≥ 21	1:1, sex, age	Vaccinated patients with MI or planned stenting (n = 145)	Unvaccinated patients with MI or planned stenting (n = 147)	Coronary ischemic events and vascular deaths
	de la Fuente2^35^	2002	Argentina	≥ 21	1:1, sex, age	Vaccinated patients with MI or planned stenting (n = 151)	Unvaccinated patients with MI or planned stenting (n = 150)	Coronary ischemic events and vascular deaths
Cohort	Thomsen^36^	2019	Denmark	≥ 65	1:1.5, sex	Vaccinated ICU survivors (n = 28,353)	Unvaccinated ICU survivors (n = 44,984)	Coronary ischemic events, stroke, and heart failure
	Hsin Wu^37^	2019	Taiwan	76 ± 6^a^	1:1, sex, age	Vaccinated patients with diagnoses of acute MI (n = 4253)	Unvaccinated patients with diagnoses of acute MI(n = 4219)	Heart failure
	Lavallée^7^	2014	Multicenter	≥ 31	1:3, sex, age	Vaccinated patients with stroke or TIA (n = 5672)	Unvaccinated patients with stroke or TIA (n = 16,901)	Coronary ischemic events, stroke, vascular deaths
	Loeb^38^	2012	Multicenter	≥ 55	1:1.5	Vaccinated patients with vascular disease or diabetes mellitus (n = 40,130)	Unvaccinated patients with vascular disease or diabetes mellitus (n = 61,621)	Cardiovascular death, nonfatal myocardial infarction, and nonfatal stroke
	de la Fuente^39^	2004	Argentina	≥ 65	1:1, sex, age	Vaccinated patients with myocardial infarction (n = 114)	Unvaccinated patients with myocardial infarction (n = 108)	Coronary ischemic events
Case–control	MacIntyre^40^	2013	Australia	≥ 40	1:1	Patients admitted with an acute myocardial infarction (n = 275)	Patients in orthopedic or ophthalmic outpatient clinics (n = 284)	Coronary ischemic events
	Heckbert^41^	2006	USA	≥ 72	1:2.5, sex, age	Patients with myocardial infarction (n = 750)	Patients without myocardial infarction (n = 1735)	Coronary ischemic events
	Barlas^42^	2000	USA	63 ± 12^a^	1:1, sex, age	CHD Patients With New MI (n = 109)	CHD Patients Without New MI (n = 109)	Coronary ischemic events
	Beahm^43^	2004	USA	70^a^	1:1.5, sex, age	Patients with myocardial infarction (n = 335)	Patients with bone fractures (n = 199)	Coronary ischemic events
	Hsien Chiang^44^	2017	Taiwan	≥ 65	1:1, sex, age	Patients who hospitalized with MACE (n = 80,363)	Patients with no previous MACE (n = 80,363)	Coronary ischemic events and stroke
	Siriwardena^25^	2010	UK	≥ 40	1:4, sex, age	Patients with acute myocardial infarction (n = 16,012)	Patients without acute myocardial infarction (n = 62,694)	Coronary ischemic events

^a^These studies did not report age range, so the mean (± standard deviation) was reported.

### RCT studies

**Figure 2:**
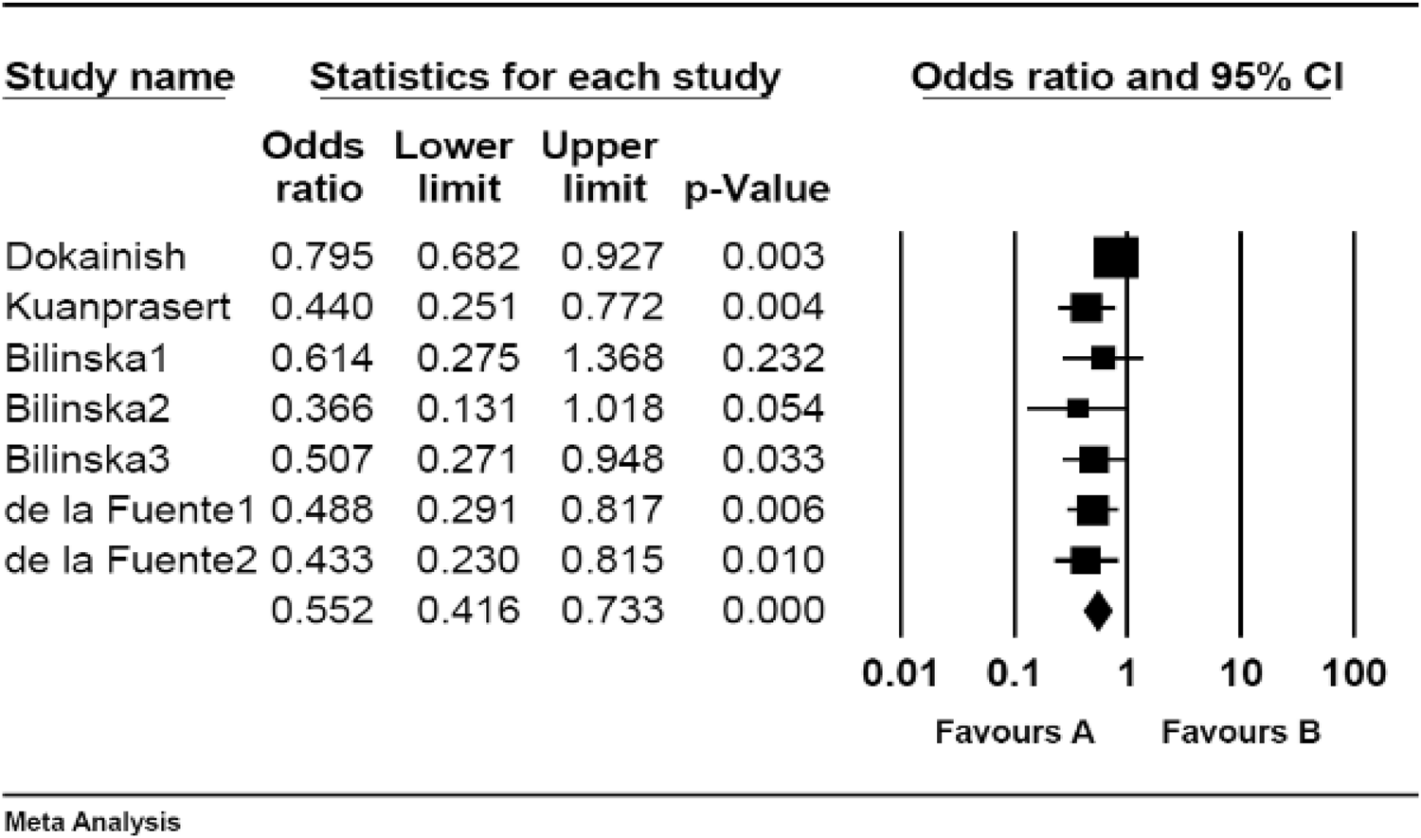
Pooled RR for RCT studies.

Six RCT studies investigated the risk of CVD after influenza vaccination. As shown in Table 2 and Fig. 2, the RR of developing CVD events after influenza vaccination was 0.55 (95% CI 0.41–0.73). Thus, influenza vaccination significantly decreased the risk of developing CVD events (P-value = 0.00).

**Table 2:** Pooled RR or OR for included studies.

Type of study	Number of studies	Pooled RR or OR (95% CI)	*p*-value	Heterogeneity test		Publication bias (*p*-value)
				I^2^ (%)	*p*-value	
RCT	6	0.55 (0.41–0.73)	0.00	50	0.06	1.00
Cohort	5	0.89 (0.77–1.04)	0.15	88	0.00	0.80
Case–control	6	0.70 (0.57–0.86)	0.00	98	0.00	1.00

### Cohort studies

**Figure 3:**
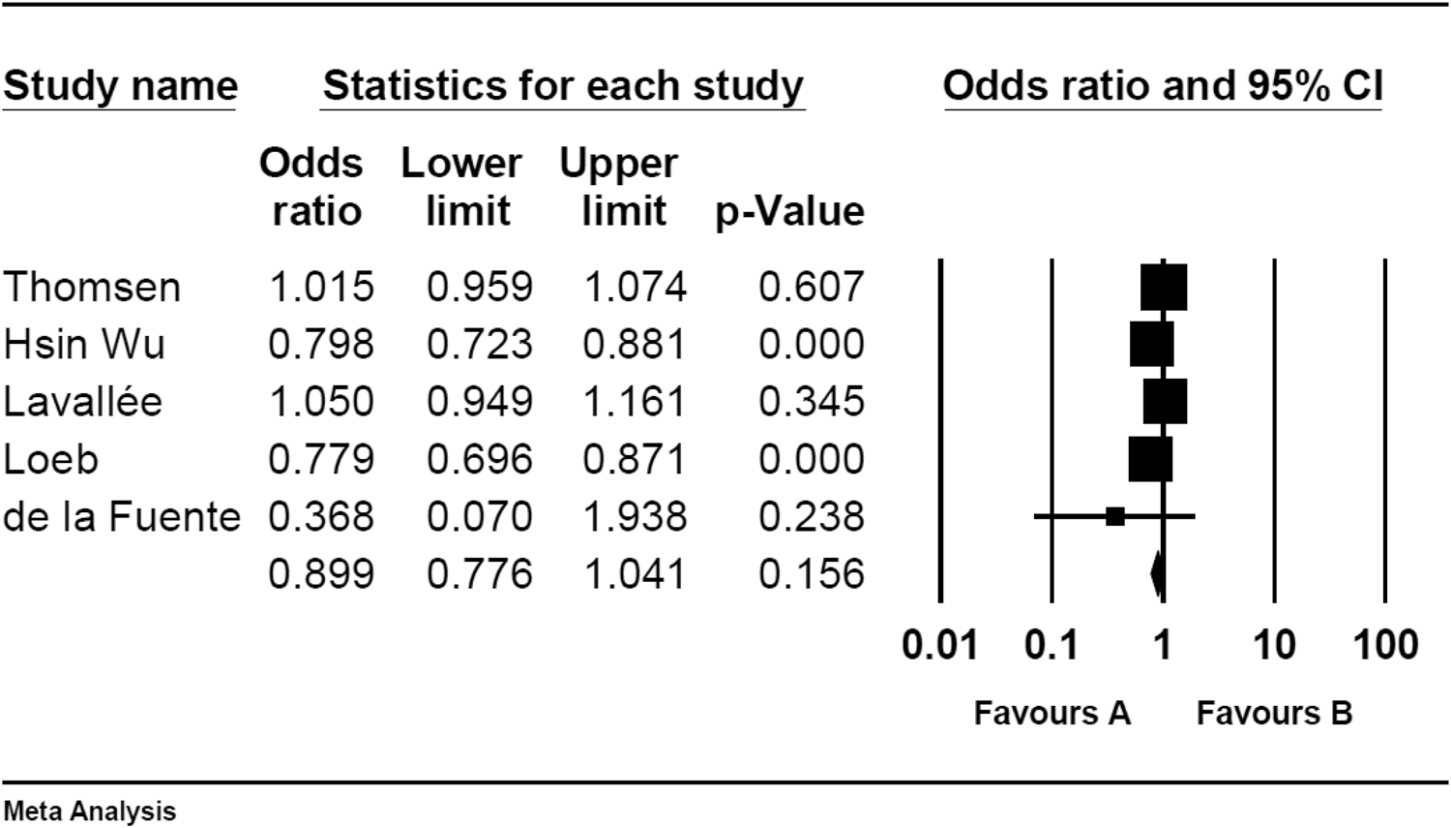
Pooled OR for cohort studies.

Six case–control studies investigated the risk of developing CVD after influenza vaccination. The pooled OR of decreasing CVD events after influenza vaccination was 0.89 (95% CI 0.77–1.04 (Table 2 and Fig. 3).

### Case–control studies

**Figure 4:**
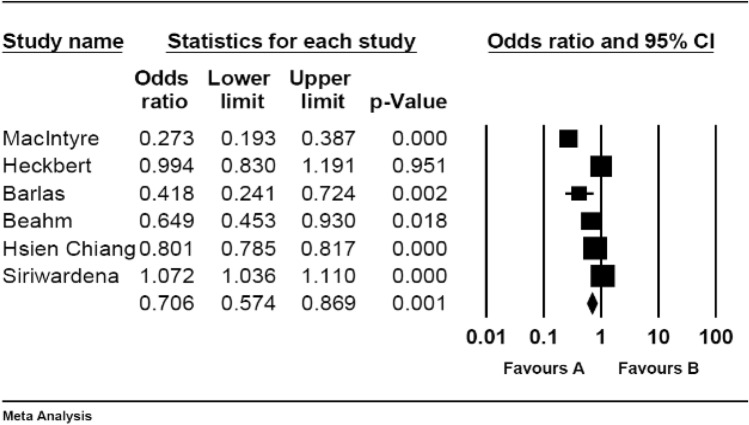
Pooled OR for case–control studies.

The pooled OR of developing CVD after influenza vaccination by pooling case–control studies was 0.70 (95% CI 0.57–0.86) (Table 2 and Fig. 4). In these studies, vaccination against influenza significantly decreased the risk of developing CVD (P-value = 0.00).

## Discussion

Our meta-analyses of RCT and case–control studies showed a significant decrease in the risk of developing CVD events as a result of influenza vaccination. Also, the analysis of cohort studies represented a decrease in the risk of CVDs, whereas it was not statistically significant.

CVD is the leading cause of mortality and morbidity around the world^13^. CVD is more prevalent in the cold seasons, which coincides with influenza outbreaks occurring during the same period. Low temperatures and influenza infection both appear to exacerbate the symptoms of CVD, so that hospitalization due to myocardial infarction also increases in winter with outbreaks of influenza^14,15^.

During influenza seasons in the United States, 54–70% of hospitalizations and 71–85% of deaths occurred among adults aged more than 65 annually were reported to be due to influenza infection^16^. Some of the mechanisms by which influenza infection affects CVD events include endothelial dysfunction, development of acute obstruction of coronary arteries by causing hypoxia and tachycardia, inducement of pro-inflammatory cytokines, disruptive effects on anticoagulant mechanisms, and pro-coagulant activity^17–19^. Influenza infection by triggering the thrombosis of a pre-existing atherosclerotic plaque can lead to acute coronary occlusion and subsequent AMI^20^. Additionally, inflammatory cell infiltration due to the infection may cause rupture of vulnerable plaques which in turn can result in acute coronary syndromes^21^.

Researchers have recently indicated that influenza vaccines may be a useful strategy to reduce CVD events, especially in high-risk groups who are prone to undergo more severe complications^22^. Gurfinkel and colleagues reported a significant reduction of the risk of death and ischemic events in patients suffering from CVD following a single intramuscularly dose of influenza vaccination^23^. In the case of AMI, the effectiveness of influenza vaccination was reported to be between 19 and 45% based on relevant studies^24,25^. The results of Wu et al., the study confirmed the beneficial role of influenza vaccination in reducing the risk of CVDs in elderly patients with previous myocardial infarction^26^.

Calderia and colleagues conducted a systematic review and meta-analysis of self-controlled case series about the risk of MI associated with Influenza infection as well as the effects of vaccination .they concluded that the Influenza vaccination was safe regarding the short-term risk for MI^27^. Their results were in line with our study.

Barens and colleagues in 2015 evaluated the effect of the influenza vaccination on AMI in a meta-analysis of case–control studies. They reported a significant association between recent influenza infection and AMI as well as the influenza vaccine effectiveness for secondary prevention of AMI^28^. The results of the current study also showed the protective role of influenza vaccination on CVD events.

In recent years, several studies have been published to evaluate influenza vaccination to reduce the risk of CVD events. Some of these studies have shown that the vaccine is effective and safe in reducing the risk of MI, while some have not achieved this result and have not considered vaccination as a useful way to reduce the risk of MI. Of course, several factors, including the inclusion and exclusion criteria of patients, the geographical area, genetic background or underlying condition, and other factors can affect the results of different studies.

However, meta-analysis studies that have reviewed and summarized existing research have generally shown the effectiveness of influenza vaccination in reducing the risk of cardiovascular disease. The results of the present study, after reviewing the pros and cons, finally confirm these beneficial effects.

Although vaccination is a simple, inexpensive, and affordable way to control many infections and their complications, especially in people with predisposing factors such as CVDs, this vaccination program has not received much attention in many countries^29,30^. Additionally, the rate of vaccination is as low as about 30% in groups younger than 65 years old that are at high risk for cardiovascular disease and its associated complications^17,31^. Accordingly, we recommend that physicians be given sufficient information regarding the protective effect of influenza vaccination and encourage patients with CVD, particularly those in high-risk age groups such as the elderly, who are more likely to develop severe complications from infections, to receive an annual flu vaccine, especially before the onset of cold seasons.

Our study had some limitations. First, only studies in English were included, which may have caused important studies to be missed. Second, heterogeneity exists among the included studies, which may limit our interpretation of the association of influenza vaccination with a lower risk of cardiovascular events. Third, our included studies were limited to some Western and Asian countries, whereas differ notably about stroke incidence. Finally, the potential influence of age, sex, and time of vaccination could not be analyzed because of the limited information obtained from the studied articles.

In conclusion, the results of this meta-analysis do support the protective role of influenza vaccination on CVD events. Health authorities may develop evidence-based preventive strategies to offer influenza vaccination in patients with CVDs.

## Supplementary information

Supplementary Information
