# Supplementary material for: Protective effect of influenza vaccination on cardiovascular diseases: a systematic review and meta-analysis
Source: Sci Rep. 2020 Nov 26;10:20656. doi: 10.1038/s41598-020-77679-7 (PMC7692477; doi:10.1038/s41598-020-77679-7)
Supplement: Supplementary file 1 — Supplementary Information [file 41598_2020_77679_MOESM1_ESM.docx]

**Protective effect of influenza vaccination on cardiovascular diseases:**

**A systematic review and meta-analysis**

Moein Zangiabadian^1^, Seyed Aria Nejadghaderi^1^, Mehdi Mirsaeidi^2*^, Bahareh Hajikhani^3^, Mehdi Goudarzi^3^, Hossein Goudarzi^3^, Masoud Mardani^4^ and Mohammad Javad Nasiri^3*^

^1^ School of Medicine, Shahid Beheshti University of Medical Sciences, Tehran, Iran

^2^ Department of Medicine, Division of Pulmonary, Critical Care, Sleep and Allergy, University of Miami, Coral Gables, Florida, USA

^3^ Department of Microbiology, School of Medicine, Shahid Beheshti University of Medical Sciences, Tehran, Iran

^4^ Infectious Diseases and Tropical Medicine Research Center, Shahid Beheshti University of Medical Sciences, Tehran, Iran

**Corresponding authors:**

Mohammad Javad Nasiri, PhD

Email: [mj.nasiri@hotmail.com](mailto:mj.nasiri@hotmail.com)

Mehdi Mirsaeidi, MD, MPH

Email: [msm249@miami.edu](mailto:msm249@miami.edu)

Table S1. Search strategy for PubMed/Medline.

| **#** | **Search terms** | **Results (Search date: November 23, 2019)** |
| --- | --- | --- |
| 1 | (Myocardial Infarction[MeSH Terms]) OR Myocardial Infarction[Title/Abstract] OR Myocardial infarction [Title/Abstract] OR Cardiovascular[Title/Abstract] OR Atherosclerosis[Title/Abstract] OR Atrial fibrillation[Title/Abstract] OR Stroke[Title/Abstract] OR Coronary[Title/Abstract] | 241,189 |
| 2 | (Influenza Vaccines[MeSH Terms]) OR Influenza Vaccine[Title/Abstract] OR Influenza[Title/Abstract] OR Flu[Title/Abstract] OR Respiratory infection[Title/Abstract] | 24,052 |
| 3 | #1 AND #2 | 76 |
